# Perceived discrimination, trust in physicians, and their associations with ovarian cancer mortality among women in the African American Cancer Epidemiology Study

**DOI:** 10.1007/s10552-025-01995-4

**Published:** 2025-05-06

**Authors:** Lindsay J. Collin, Courtney E. Johnson, Maxwell Akonde, Mary Kan, Elisa V. Bandera, Lauren C. Peres, Bo Qin, Michele L. Cote, Anthony Alberg, Edward S. Peters, Theresa A. Hastert, Joellen M. Schildkraut

**Affiliations:** 1 Department of Epidemiology, Rollins School of Public Health, Emory University, 1518 Clifton Rd NE, Atlanta, GA 30322 USA; 2 Department of Epidemiology and Biostatistics, Arnold School of Public Health, University of South Carolina, Columbia, SC USA; 3 Cancer Epidemiology and Health Outcomes, Rutgers Cancer Institute, New Brunswick, NJ USA; 4 Department of Cancer Epidemiology, Moffitt Cancer Center, Tampa, FL USA; 5 Simon Comprehensive Cancer Center, Indiana University, Indianapolis, IN USA; 6 Department of Epidemiology, UNMC College of Public Health, Omaha, NE USA; 7 Cancer Prevention and Control Program, Fred & Pamela Buffett Cancer Center, Omaha, NE USA; 8 Department of Oncology, Wayne State University School of Medicine, Detroit, MI USA; 9 Population Studies and Disparities Research Program, Karmanos Cancer Institute, Detroit, MI USA

**Keywords:** Ovarian cancer, Cancer health disparities, Medical mistrust, Discrimination

## Abstract

**Purpose:**

Black women are 30% more likely to die of ovarian cancer than White women. Discrimination may affect cancer health disparities through pathways including socioeconomic disadvantage, chronic stress, and access to care. In this study, we evaluated associations of discrimination and trust in physicians with all-cause mortality among Black women with ovarian cancer.

**Methods:**

Using data from the African American Cancer Epidemiology Study (AACES), we included 592 Black ovarian cancer patients who completed an interview. Discrimination and trust in physicians were measured using the Everyday Discrimination, Major Experiences of Discrimination, and Trust in Physicians scales, respectively. We used Cox proportional hazard models to compute multivariable-adjusted hazard ratios (HR) and 95% confidence intervals (CIs) associating everyday discrimination, major experiences of discrimination, and trust in physicians with all-cause mortality.

**Results:**

Approximately 43% reported experiencing at least one major experience of discrimination, 16% reported high everyday experiences of discrimination, and the median trust in physician score was 35. The association between higher experiences of everyday discrimination was HR = 0.84 (95% CI: 0.63, 1.11), compared with low experiences of everyday discrimination. We observed that more major experiences of discrimination had 1.25-times the mortality rate compared with low experiences of major discrimination (95% CI: 0.84, 2.20). Higher trust in physicians was associated with slightly lower mortality rates (HR = 0.91, 95% CI: 0.74, 1.14).

**Conclusion:**

We observed complexities in the relationships of everyday discrimination, major experiences of discrimination, and trust in physicians with mortality among Black women with ovarian cancer. Future work to understand the these relationships is likely warranted.

**Supplementary Information:**

The online version contains supplementary material available at 10.1007/s10552-025-01995-4.

## Introduction

Ovarian cancer is a rare disease, with approximately 20,000 new diagnoses in the US in 2024; however, it is the sixth leading cause of cancer-related mortality among women in the US [1]. There are pronounced and well-documented racial disparities in ovarian cancer mortality, wherein Black women are nearly 30% more likely to die from their disease compared with White women, despite having a lower incidence of ovarian cancer [2, 3]. Factors that contribute to these disparities are complex and multifactorial, with access to care, disease severity, and socioeconomic factors potentially explaining some of the observed disparity [4–7]. In multivariable-adjusted analyses that account for health insurance, socioeconomic status, and demographic and clinical characteristics, the racial disparities in mortality persist [2–4, 8, 9]. Racial discrimination has been proposed as another potential contributor to the observed disparity; however, it has not been evaluated in the context of ovarian cancer [10].

Racial discrimination is thought to impact cancer health disparities through distinct pathways [10]. One is through discrimination’s effect on socioeconomic inequities which can influence access to care and quality of care received, ultimately impacting timeliness and quality of care for newly diagnosed cancer patients. Another is through chronic stress, which can lead to inflammation [11]. Chronic inflammation has been linked to changes in the tumor immune microenvironment [12–16], as well as survival among ovarian cancer patients. These pathways have not been explored in detail in the context of cancer health disparities. Some studies have reported lower breast, cervical, and colorectal cancer screening rates among individuals reporting high perceived discrimination, and one study reported a lower uptake of genetic testing among ovarian cancer patients who reported discrimination in their employment [17–21]. Trust in physicians is another measure that can influence a patient’s engagement in cancer treatment and follow-up care, potentially impacting cancer survival. No studies have looked at these two measures in relation to ovarian cancer mortality.

Previous work in the African American Cancer Epidemiology Study (AACES) of ovarian cancer patients reported that trust in physicians was not associated with prolonged symptom duration prior to an ovarian cancer diagnosis, but everyday discrimination was associated with prolonged symptom duration, which may reflect preventable delays in diagnosis [22]. In this study, we evaluated associations of perceived discrimination, including both everyday discrimination and major experiences of discrimination, and trust in physicians with all-cause mortality in the AACES cohort.

## Methods

### Study population

AACES is a multisite, population-based case control study of ovarian cancer patients who self-identify as Black or African American. For the purposes of this study, the cohort of ovarian cancer patients (cases) with follow-up information were included. Study sites include Alabama, Georgia, Illinois, Louisiana, metropolitan Detroit, Michigan, North Carolina, New Jersey, Ohio, South Carolina, Tennessee, and Texas. Institutional review board approval was obtained from all participating sites. Cases were identified via rapid case ascertainment through state or Surveillance, Epidemiology, and End Results (SEER) cancer registries and hospital gynecologic oncology departments, and enrolled between December 2010 and December 2015. Self-identified Black women who were diagnosed with histologically confirmed invasive epithelial ovarian cancer between the ages of 20 and 79 and who were able to complete an interview in English were eligible to participate in the study. AACES participants completed computer-assisted telephone interviews. A short version was offered to women who would have otherwise refused to participate. Additional information about the AACES study population has been published previously [23, 24].

### Primary exposures

Everyday discrimination was captured using the 5-question version of the Everyday Discrimination Scale [25]. Each question assesses the day-to-day occurrences of unfair treatment (e.g., “How often are you treated with less courtesy or respect than other people?”), and response options offer a six-point scale: 1 = almost every day, 2 = at least once a week, 3 = a few times a month, 4 = a few times a year, 5 = less than once a year, and 6 = never. Responses to each item were reverse-coded and averaged across all five items to create an overall everyday experiences of discrimination score ranging from 1 to 6, where lower scores would indicate rare or never experiencing everyday discrimination and higher scores would indicate greater frequency of daily experiences of discrimination. Supplemental Table 1 shows the five questions asked, with the distribution of responses. For the analyses, everyday discrimination was categorized into low (1), medium (> 1–2), and high (> 2) based on the distribution of the score in our study population where more than half of the responses were “6 = never”.

**Table 1:** Description of the demographic and clinical characteristics of the AACES Cohort

Characteristics	*n* = 592
	Median (IQR)
Age at diagnosis (years)	58 (51, 66)
Follow-up (years)	4.8 (2.4, 9.6)
	*n* (%)
Vital status	
Dead	390 (66%)
Alive	202 (34%)
Diagnosis year	
2010	4 (0.7%)
2011	107 (18%)
2012	148 (25%)
2013	138 (23%)
2014	125 (21%)
2015	70 (12%)
Stage	
I	131 (24%)
II	52 (9.4%)
III	320 (58%)
IV	48 (8.7%)
Unknown	41
Histotype	
HGSC	397 (67%)
Mucinous	29 (4.9%)
Clear cell	23 (3.9%)
Carcinosarcoma	18 (3.0%)
LGSC	17 (2.9%)
Endometrioid	57 (9.6%)
Other	45 (7.6%)
Unknown	6
Insurance type	
Private	189 (32%)
Uninsured	50 (8.4%)
Medicaid	122 (21%)
Medicare	123 (21%)
Other	30 (5.1%)
Private + Medicare	25 (4.2%)
Unknown	53
Marital status	
Married	195 (33%)
Single	142 (24%)
Divorced	166 (28%)
Widowed	89 (15%)
Charlson comorbidity index	
0	216 (36%)
1	136 (23%)
2	99 (17%)
3 +	141 (24%)
Smoking status	
Current	102 (17%)
Former	163 (28%)
Never	327 (55%)
Alcohol use (Yes)	235 (49%)
Unknown	109
Physical activity (Yes)	133 (25%)
Unknown	52
Education	
College	112 (19%)
Grad school	72 (12%)
HS/GED	302 (51%)
Some college	106 (18%)
Family income	
< $10,000	115 (21%)
$10,000- < $25,000	125 (23%)
$25,000- < %50,000	132 (24%)
$50,000- < $75,000	79 (15%)
$75,000-$100,000	48 (8.9%)
> $100,000	32 (5.9%)
Missing	61
Social support	
Q4 (highest)	124 (23%)
Q3	143 (27%)
Q2	49 (9.1%)
Q1 (lowest)	222 (41%)
Unknown	54
Region	
South	396 (67%)
North	138 (23%)
Texas	58 (10%)
Yost index	
1 (Highest SES)	185 (31%)
2	91 (15%)
3	61 (10%)
4	60 (10%)
5 (Lowest SES)	24 (4.1%)
Unknown	171

*HGSC* high-grade serous ovarian cancer, *LGSC* low-grade serous ovarian cancer, *SES* socioeconomic status

Major experiences of discrimination was captured by the Major Experiences of Discrimination Scale [25], which includes questions on experience of discrimination within the domains of job promotion/hiring, police encounters, education, housing, and loans. They were also asked to recall when the event occurred (*i.e.,* within the last week, month, year, or more than a year ago) if they had experienced discrimination in one of the domains. In Supplemental Table 2 we report the specific questions asked and the distribution of yes/no responses in the AACES population. To operationalize the Major Experiences of Discrimination Scale for the analysis, a summary score was created based on the six item responses where each “no” response was assigned a score of 0 and each “yes” was assigned a score of 1 (ranged 0–6) to represent major experiences of discrimination over the lifetime. As there were relatively few participants with three or more major experiences of discrimination, we operationalized the major experiences of discrimination as a categorical variable: 0, 1–2, and ≥ 3 due to sparse data. Moreover, we examined each domain of the major experiences of discrimination individually as yes/no responses to see if any specific domain contributed to ovarian cancer mortality.

**Table 2:** Associations of Everyday Discrimination, Major Experiences of Discrimination, and Trust in Physicians with all-cause mortality in the African American Cancer Epidemiology Study (AACES) cohort

Measure	Total *n* (%)	Events *n* (%)	HR (95% CI)^1^	HR (95% CI)^2^
Everyday experiences of discrimination				
High	85 (16)	47 (13)	0.83 (0.60, 1.14)	0.87 (0.62, 1.22)
Moderate	182 (34)	121 (34)	1.04 (0.83, 1.31)	0.98 (0.76, 1.27)
Low	273 (51)	185 (52)	Ref	Ref
Dichotomized				
High	267 (49)	168 (48)	0.97 (0.79, 1.20)	0.84 (0.63, 1.11)
Low	273 (51)	185 (52)	Ref	Ref
Major experiences of discrimination				
Any	229 (43)	137	0.94 (0.85, 1.06)	0.79 (0.64, 0.98)
None	302 (57)	211	Ref	Ref
Major experiences of discrimination				
High	33 (6.2)	22 (6.3)	1.00 (0.64, 1.55)	1.26 (0.84, 2.20)
Moderate	196 (37)	114 (33)	0.75 (0.60, 0.94)	0.70 (0.55, 0.89)
Low	302 (57)	211 (72)	Ref	Ref
Domains of major experiences of discrimination				
Job fired/denied promotion				
Yes	132 (25)	70 (21)	0.84 (0.65, 1.09)	0.78 (0.59, 1.03)
No	403 (75)	266 (89)	Ref	Ref
Job (Unfairly not hired)				
Yes	73 (14)	45 (13)	1.06 (0.78, 1.44)	1.07 (0.77, 1.49)
No	463 (86)	293 (87)	Ref	Ref
Police discrimination				
Yes	31 (5.8)	18 (5.3)	0.92 (0.58, 1.46)	0.87 (0.53, 1.42)
No	507 (94)	320 (95)	Ref	Ref
Education discrimination				
Yes	83 (15)	50 (15)	0.86 (0.64, 1.16)	0.85 (0.63, 1.15)
No	457 (85)	290 (85)	Ref	Ref
Housing discrimination				
Yes	26 (4.8)	18 (5.2)	0.95 (0.60, 1.51)	1.12 (0.69, 1.79)
No	514 (95)	322 (95)	Ref	Ref
Bank discrimination				
Yes	40 (7.4)	25 (7.4)	0.94 (0.63, 1.40)	1.10 (0.73, 1.65)
No	498 (93)	314 (93)	Ref	Ref
Trust in physicians				
Trust in physicians (per 1-unit increase)	510	334	0.99 (0.98, 1.01)	0.99 (0.98, 1.01)
High trust (> 35)	292 (57)	188 (56)	0.90 (0.72, 1.11)	0.91 (0.74, 1.14)
Low trust (≤ 35)	218 (43)	146 (44)	Ref	Ref

^1^Age Adjusted

^2^Adjusted for age, stage, histotype, education, marital status, family income, social support

Trust in physicians was assessed from the Trust in Physician Scale—an 11-item questionnaire with five possible responses on a Likert scale (ranging from strongly agree to strongly disagree) [26]. The scale included prompts such as the degree to which participants felt that their physicians cared about them, or if they trusted their doctor to put their medical needs above other considerations. We coded the questions such that a higher score indicates higher trust in physicians and scores were summed across the 11 questions for a range of 0–44. Based on the distribution of the scores in the study population, we dichotomized the trust in physician scale into high/low medical mistrust at the median value of 35. Supplemental Table 3 includes the questions and responses for the Trust in Physician Scale.

### Outcome

The primary outcome of interest was all-cause mortality. Vital status and follow-up information were ascertained via the National Death Index, cancer registries, the LexisNexis database, and patient contact. Follow-up time was determined from the time of enrollment/interview to the first of death or last contact (6 November 2023). The time from ovarian cancer diagnosis to the date of interview was excluded to avoid immortal person-time bias, which may be a concern if the exposures are associated with mortality before participants were able to be enrolled [27].

### Covariates

Covariates were selected based on literature review and causal diagrams and included: age at diagnosis (continuous, years), stage at diagnosis (I–IV), histotype (low-grade serous, high-grade serous, mucinous, clear cell, carcinosarcoma, endometrioid, and other), marital status (married, single, divorced, widowed), education (high school/GED, some college, college graduate, graduate school), family income (< $10,000, $10,000– < $25,000, $25,000– < $50,000, $50,000– < $75,000, $75,000–$100,000, > $100,000), insurance type (private, Medicaid, Medicare, uninsured, unknown), and social support. Social support was assessed from Likert-based responses to twelve questions identifying support mechanisms. The responses were then summed to create a summary score (ranging 0–48), and we categorized into quartiles for this analysis. Due to the small numbers of the less common histotypes (*e.g.,* clear cell, mucinous, and low-grade serous) we grouped histotypes as high-grade serous/carcinosarcoma vs. non-high-grade serous/carcinosarcoma as defined by the type II and type I designation for ovarian cancer, respectively [28, 29].

### Statistical analysis

We calculated descriptive statistics for the study population reporting frequencies and proportions or medians and interquartile ranges of the study exposures (everyday experiences of discrimination, major experiences of discrimination, and trust in physicians), sociodemographic characteristics, tumor, and clinical characteristics of the study population overall.

Cox proportional hazards regression was used to estimate the hazard ratios (HRs) and 95% confidence intervals (CI) to examine the associations between everyday discrimination, major experiences of discrimination, and trust in physicians with all-cause mortality. We reported age-adjusted associations and multivariable-adjusted associations. Variables included in the multivariable-adjusted associations were determined a priori based on subject matter knowledge and literature review and included age, stage, histotype, marital status, family income, education, and social support. In multivariable-adjusted analyses, missing data were excluded, and a complete case analysis was conducted. No hypothesis testing was performed [30–33]. All analyses were carried out in R v 4.3 (Vienna, Austria).

## Results

### Description of study population

Among the 592 ovarian cancer patients included in the AACES cohort, 67% were diagnosed with stage III/IV disease and 67% were classified as high-grade serous ovarian cancer (Table 1). The average age at diagnosis was 58 years (interquartile range [IQR] 51, 66). By the end of follow-up, 390 (66%) had died, with a median 4.8 years (IQR: 2.4, 9.6) follow-up time. The majority were either married (33%) or divorced (28%) at the time of interview. Approximately 19% had received college-level education, with the majority reporting high school or GED (51%). The distribution of family income ranged from 21% with < $10,000 to 5.9% with > $100,000, and a plurality (24%) reported an annual family income between $25,000 and $50,000.

### Everyday discrimination

Among the AACES cohort, approximately half of the cohort (51%) reported low levels (1) of everyday discrimination, and 16% of AACES participants reported high levels (> 2) of everyday discrimination (Table 2). Compared with those participants reporting low levels of everyday discrimination, neither high (HR = 0.87; 95% CI: 0.62, 1.22) nor moderate (HR = 0.98; 95% CI: 0.76, 1.27) everyday discrimination was associated with all-cause mortality. We observed a similar estimate of association in the dichotomized version of the everyday discrimination score, which combined high and moderate (compared with low) levels of everyday discrimination (HR = 0.84; 95% CI: 0.63, 1.11). We also descriptively examined which questions contributed to experiences of higher everyday discrimination. In the AACES cohort, the three questions where participants were most likely to respond to frequent (*i.e.,* weekly, or more frequently) forms of everyday discrimination included: being treated with less courtesy (4.4%), people acting like they were not smart (3.9%), and having people act like they were afraid of you (2.6%) [Table S1]. We were unable to analyze these as independent variables in the models due to the small sample size.

### Major experiences of discrimination

Relatively few participants (6.2%) reported high (≥ 3) major experiences of discrimination and 37% reported moderate (1–2) experiences of discrimination. In Table 2, we present the associations between major experiences of discrimination and mortality. Reporting at least one major experience of discrimination was associated with a lower all-cause mortality compared with reporting no major experiences of discrimination (HR = 0.79, 95% CI: 0.64, 0.98). Further stratification suggested that reporting 2 + major experiences of discrimination was associated with mortality (HR = 1.26, 95% CI: 0.84, 2.20), while reporting one was inversely associated with mortality (HR = 0.70, 95% CI: 0.55, 0.89) compared with no major experiences of discrimination. We further estimated associations between each type of major experiences of discrimination and mortality. Most individual forms of major discrimination were not associated with mortality; however, we did observe that being fired from a job or denied promotion was associated with lower mortality rates (HR = 0.78, 95% CI: 0.59, 1.03).

### Trust in physicians

Table 2 presents associations between high trust in physicians compared with low trust in physicians (cut at the median score of 35) and all-cause mortality. Comparing those with high versus low trust in physicians, in a fully adjusted model, higher trust in physicians showed slightly lower mortality rates (HR = 0.91; 95% CI: 0.74, 1.14). When we examined a per-unit increase in the trust in physician scale, the association was null.

## Discussion

In this study, we did not observe an association between everyday discrimination and all-cause mortality among Black ovarian cancer patients. In the three-level categorization of major experiences of discrimination, we observed that among individuals who experienced the most major experiences of discrimination ovarian cancer mortality was slightly higher, although the estimate was imprecise. However, comparing any versus no major experiences of discrimination, was associated with slightly lower mortality rates, which appeared to be driven by those reporting experiences of discrimination in job promotion and hiring. Finally, we observed that higher trust in physicians was associated with slightly lower mortality rates among Black ovarian cancer patients.

Previous studies have shown that experiences of racial discrimination—both everyday and major experiences of discrimination—contribute to higher mortality rates among marginalized communities. A study using the Multi-Ethnic Study of Atherosclerosis (MESA) cohort data reported that individuals experiencing more instances of discrimination had 1.06-times the all-cause mortality rate and 1.15-times the cardiovascular disease-specific mortality rate, with more pronounced associations among Black participants in the study, but results were attenuated among women (all racial and ethnic groups) [34]. We observed similar point estimates in the three-level categorization of major experiences of discrimination; however, the estimates were imprecise. Evidence on perceived discrimination in relation to cancer outcomes is limited. Studies have reported that perceived discrimination is associated with an increased risk of breast cancer, among all women, but no study has looked at discrimination with respect to cancer mortality [18–20]. A recent study using data from the Detroit Research on Cancer Survivors cohort reported that experiences of everyday discrimination were associated with frailty among Black cancer survivors [35]. In the current study, we observed slightly lower mortality rates associated with any compared with no major experiences of discrimination, but with some evidence that those with two or more experiences of major discrimination may have slightly higher mortality rates. In our study, the proportion reporting frequent major experiences of discrimination was low (~ 6%), which may contribute to the heterogeneity in results comparing our study with other studies.

Certain specific examples of major experiences of discrimination (e.g., being unfairly fired or denied promotion) may be more common among women in professional occupations compared with those who did not report employment-related discrimination. In our study population, the most common major experience of discrimination was related to being denied or hired for a job (25%). This represents a potentially distinct population than the broader ovarian cancer patient population as those who are in the position to be denied job promotion or hired are more likely to be moving towards career advancement and are more likely to be in professional occupations [36]. As a result, our observed protective association between major experiences of discrimination and all-cause mortality may reflect residual confounding my socioeconomic status, even though we adjusted for family income and education in the models. To evaluate whether this might contribute to the overall observed protective association between major experiences of discrimination and mortality, we looked at the distribution of different demographic characteristics by those who responded yes/no to this prompt (Table S4). Women who reported discrimination employment discrimination were younger (56 vs 59 years), a higher proportion were divorced (38% vs 25%), and more were likely to have completed college (26% vs. 17%) compared with those who did not report being unfairly fired or denied promotion. These differences could potentially indicate residual confounding or lack of exchangeability of the two groups [37]. Future studies with larger samples sizes may benefit from exploration of effect modification by socioeconomic factors.

The relationship between discrimination and socioeconomic factors was evident in the current analysis and indicates a complex relationship. We observed that those who reported major experiences of discrimination were more likely to have a higher family income. On the other hand, those who reported more everyday experiences of discrimination were more likely to report a lower family income. The study by Lawrence et al. using data from MESA similarly observed that those reporting any major experiences of discrimination were more likely to have a college degree or higher. The complexity between components of socioeconomic status and everyday discrimination, major experiences of discrimination, and trust in physician likely warrants further exploration in future work.

Previous studies have indicated that trust in physicians influences a patient’s willingness to engage in healthcare, and adhere to treatment guidelines and follow-up care [38–40]. In our study, we did not observe an association between trust in physicians and mortality, but the estimate was imprecise. Individuals with lower physician trust may be more likely to delay treatment or appointments, which could negatively impact outcomes. Previous studies on trust in physicians and cancer outcomes have indicated that higher trust in physicians facilitates improved treatment decision making, treatment adherence, and outcomes [38]. A previous study among breast cancer patients reported that trust in physicians was related to trust in the healthcare system, which was also related to treatment adherence [39]. This study reported a mean trust in physician score of 29 in a sample of approximately 2,000 breast cancer patients, which was less than the mean in our study sample (mean = 34), but their study included Black, Hispanic, and White patients, which may have contributed to the differences. Ultimately, most studies that have examined the relationship of trust in physicians and cancer outcomes in detail, have indicated that it influences mortality through treatment adherence [40], which we were unable to examine in this study directly. We categorized trust in physicians based on the median of the distribution, whereas the study among breast cancer patients used tertiles of the distribution. We repeated our analyses using tertiles of the distribution, but due to small numbers, were unable to estimate associations with reliable precision, and the associations were similar to the results using trust in physicians dichotomized at the median.

This study is not without limitations. First, although this study is the largest cohort of Black women with ovarian cancer, we were limited by the sample size across some categories of discrimination and trust in physicians, which contributed to imprecision in the estimates of association. Second, the AACES study population recruited ovarian cancer patients near the time of diagnosis, often after completion of their primary treatment. Patients who were sicker at the time of diagnosis or who had more advanced disease would be more likely to decline to participate or pass away before the study team could make contact. This could contribute to selection bias, leading to participation of healthier individuals, and our estimates may not generalize to the broader population of Black ovarian cancer patients. Third, both trust in physicians and the two measures of discrimination were assessed via self-report through interviewer-assisted interviews. This approach may influence how participants responded to the prompts and would lead to misclassification in our observed estimates if participants responded differently than they would have without an interview assistant present. Finally, although we adjusted for key demographic and clinical characteristics, trust in physicians and discrimination are likely correlated with factors that are also associated with survival among ovarian cancer patients that we were unable to account for, leading to the possibility of residual confounding by unknown factors.

In conclusion, in this study, we observed that experiences of major discrimination events were associated with slightly lower mortality rates. However, that further investigation into the individual components of major experiences of discrimination suggested that these results were driven by individuals experiencing discrimination in job promotion or hire. We did not observe an association between everyday discrimination and mortality. Higher trust in physicians was associated with slightly lower mortality rates. Our study results highlight that these relationships between experiences of both types of discrimination and cancer outcomes are complex. Future research to disentangle these relationships may be warranted in a larger cohort of patients.

## Supplementary Information

Below is the link to the electronic supplementary material.

Supplementary file1 (DOCX 33 KB)

## Data Availability

The datasets generated during the current study for analysis are not publicly available due to patient confidentiality but can be made available through proper approval from relevant Institutional Research Boards and the AACES investigative team.
