# Supplementary material for: Perceived discrimination, trust in physicians, and their associations with ovarian cancer mortality among women in the African American Cancer Epidemiology Study
Source: Cancer Causes Control. 2025 May 6;36(10):1069–77. doi: 10.1007/s10552-025-01995-4 (PMC12578685; doi:10.1007/s10552-025-01995-4)
Supplement: Supplementary file 1 — Supplementary file1 (DOCX 33 KB) [file 10552_2025_1995_MOESM1_ESM.docx]

**Title:** Perceived discrimination, trust in physicians, and their associations with ovarian cancer mortality among women in the African American Cancer Epidemiology Study

**Author List:** Lindsay J. Collin,^1^ Courtney E. Johnson,^1^ Maxwell Akonde,^2^ Mary Kan,^1^ Elisa V. Bandera,^3^ Lauren C. Peres,^4^ Bo Qin, ^3^ Michele L. Cote,^5^ Anthony Alberg,^2^ Edward S. Peters, ^6,7^ Theresa A. Hastert,^8, 9^ Joellen M. Schildkraut^1^

Table S1: Distribution of patient responses to Everyday Discrimination Scale in the African American Cancer Epidemiology Study (AACES) population

| **Everyday Discrimination** | **N = 592**^1^ |
| --- | --- |
| **How often are you treated with less courtesy or respect than other people?** |  |
| Almost everyday | 16 (2.7%) |
| At least once a week | 8 (1.4%) |
| A few times a month | 19 (3.2%) |
| A few times a year | 43 (7.3%) |
| Less than once a year | 89 (15.0%) |
| Never | 365 (61.7%) |
| Missing | 52 (8.8%) |
| **How often do you receive poorer service than other people at restaurants or stores?** |  |
| Almost everyday | 0 (0.0%) |
| At least once a week | 5 (0.8%) |
| A few times a month | 13 (2.2%) |
| A few times a year | 62 (10.5%) |
| Less than once a year | 95 (16.0%) |
| Never | 363 (61.3%) |
| Missing | 54 (9.1%) |
| **How often do people act as if they think you are not smart?** |  |
| Almost everyday | 12 (2.0%) |
| At least once a week | 9 (1.5%) |
| A few times a month | 23 (3.9%) |
| A few times a year | 50 (8.4%) |
| Less than once a year | 84 (14.2%) |
| Never | 360 (60.8%) |
| Missing | 54 (9.1%) |
| **How often do people act as if they are afraid of you?** |  |
| Almost everyday | 9 (1.5%) |
| At least once a week | 5 (0.8%) |
| A few times a month | 12 (2.0%) |
| A few times a year | 25 (4.2%) |
| Less than once a year | 24 (4.1%) |
| Never | 464 (78.4%) |
| Missing | 53 (8.9%) |
| **How often are you threatened or harassed?** |  |
| Almost everyday | 2 (0.3%) |
| At least once a week | 4 (0.7%) |
| A few times a month | 5 (0.8%) |
| A few times a year | 12 (2.0%) |
| Less than once a year | 28 (4.7%) |
| Never | 488 (82.4%) |
| Missing | 53 (8.9%) |
| ^1^n (%) | |

**Table S2:** Distribution of responses to Major Experiences of Discrimination Scale in the African American Cancer Epidemiology Study (AACES) participants

| **Major Experiences of Discrimination** | **N = 592**^1^ |
| --- | --- |
| **Unfairly fired from a job or unfairly denied a promotion** |  |
| Yes | 132 (22.3%) |
| No | 403 (68.1%) |
| Missing | 57 (9.6%) |
| **Unfairly not hired for a job** |  |
| Yes | 73 (12.3%) |
| No | 463 (78.2%) |
| Missing | 56 (9.4%) |
| **Unfairly stopped, searched, questioned, physically threatened, or abused by the police** |  |
| Yes | 31 (5.2%) |
| No | 507 (85.6%) |
| Missing | 54 (9.1%) |
| **Unfairly discouraged by a teacher or advisor from continuing your education** |  |
| Yes | 83 (14.0%) |
| No | 457 (77.2%) |
| Missing | 52 (8.8%) |
| **Unfairly prevented from moving into a neighborhood because the landlord or realtor refused to sell or rent you a house or apartment** |  |
| Yes | 26 (4.4%) |
| No | 514 (86.8%) |
| Missing | 52 (8.8%) |
| **Unfairly denied a bank loan** |  |
| Yes | 40 (6.8%) |
| No | 498 (84.1%) |
| Missing | 54 (9.1%) |
| ^1^n (%) | |

**Table S3:** Distribution of patient responses to the Trust in Physicians scale in the African American Cancer Epidemiology Study (AACES) participants

| **Trust in Physicians** | **N = 592**^1^ |
| --- | --- |
| **I doubt that my doctor really cares about me as a person** |  |
| Strongly Agree | 13 (2.2%) |
| 1 | 53 (9.0%) |
| 2 | 32 (5.4%) |
| 3 | 235 (39.7%) |
| Strongly Disagree | 175 (29.6%) |
| Missing | 84 (14.2%) |
| **My doctor is usually considerate of my needs and puts them first** |  |
| Strongly Disagree | 11 (1.9%) |
| 1 | 36 (6.1%) |
| 2 | 30 (5.1%) |
| 3 | 273 (46.1%) |
| Strongly Agree | 159 (26.9%) |
| Missing | 83 (14.0%) |
| **I trust my doctor so much I always try to follow his/her advice** |  |
| Strongly Disagree | 8 (1.4%) |
| 1 | 33 (5.6%) |
| 2 | 58 (9.8%) |
| 3 | 281 (47.5%) |
| Strongly Agree | 129 (21.8%) |
| Missing | 83 (14.0%) |
| **If my doctor tells me something is so, then it must be true** |  |
| Strongly Agree | 14 (2.4%) |
| 1 | 102 (17.2%) |
| 2 | 102 (17.2%) |
| 3 | 236 (39.9%) |
| Strongly Disagree | 55 (9.3%) |
| Missing | 83 (14.0%) |
| **I sometimes distrust my doctor's opinion and would like a second one** |  |
| Strongly Agree | 26 (4.4%) |
| 1 | 141 (23.8%) |
| 2 | 51 (8.6%) |
| 3 | 231 (39.0%) |
| Strongly Disagree | 60 (10.1%) |
| Missing | 83 (14.0%) |
| **I trust my doctor's judgments about my medical care** |  |
| Strongly Disagree | 9 (1.5%) |
| 1 | 38 (6.4%) |
| 2 | 36 (6.1%) |
| 3 | 318 (53.7%) |
| Strongly Agree | 108 (18.2%) |
| Missing | 83 (14.0%) |
| **I feel my doctor does not do everything he/she should do for my medical care** |  |
| Strongly Disagree | 23 (3.9%) |
| 1 | 74 (12.5%) |
| 2 | 40 (6.8%) |
| 3 | 285 (48.1%) |
| Strongly Agree | 87 (14.7%) |
| Missing | 83 (14.0%) |
| **I trust my doctor to put my medical needs above all other considerations when treating my medical problems** |  |
| Strongly Disagree | 9 (1.5%) |
| 1 | 39 (6.6%) |
| 2 | 41 (6.9%) |
| 3 | 323 (54.6%) |
| Strongly Agree | 97 (16.4%) |
| Missing | 83 (14.0%) |
| **My doctor is a real expert in taking care of medical problems like mine** |  |
| Strongly Disagree | 15 (2.5%) |
| 1 | 60 (10.1%) |
| 2 | 59 (10.0%) |
| 3 | 277 (46.8%) |
| Strongly Agree | 98 (16.6%) |
| Missing | 83 (14.0%) |
| **I trust my doctor to tell me if a mistake was made about my treatment** |  |
| Strongly Disagree | 9 (1.5%) |
| 1 | 65 (11.0%) |
| 2 | 61 (10.3%) |
| 3 | 288 (48.6%) |
| Strongly Agree | 85 (14.4%) |
| Missing | 84 (14.2%) |
| **I sometimes worry that my doctor may not keep the information we discuss totally private** |  |
| Strongly Disagree | 3 (0.5%) |
| 1 | 19 (3.2%) |
| 2 | 38 (6.4%) |
| 3 | 305 (51.5%) |
| Strongly Agree | 143 (24.2%) |
| Missing | 84 (14.2%) |
| **In general, I trust my doctor to give me the best possible health care** |  |
| Strongly Disagree | 9 (1.5%) |
| 1 | 42 (7.1%) |
| 2 | 21 (3.5%) |
| 3 | 146 (24.7%) |
| Strongly Agree | 290 (49.0%) |
| Missing | 84 (14.2%) |
| ^1^n (%) | |

| **Table S4**: Distribution of population characteristics by response to those reporting discrimination in job hiring/firing | | |
| --- | --- | --- |
| **Characteristics** | **No, N = 381** | **Yes, N = 123** |
| Diagnosis Year |  |  |
| 2010 | 2 (0.5%) | 1 (0.8%) |
| 2011 | 74 (19%) | 23 (19%) |
| 2012 | 95 (25%) | 28 (23%) |
| 2013 | 86 (23%) | 29 (24%) |
| 2014 | 85 (22%) | 30 (24%) |
| 2015 | 39 (10%) | 12 (9.8%) |
| Age at Diagnosis | 59 (52, 67) | 56 (50, 62) |
| Follow-up (years) | 4.5 (2.4, 9.4) | 5.5 (2.6, 10.6) |
| Vital Status |  |  |
| 1 | 259 (68%) | 70 (57%) |
| 0 | 122 (32%) | 53 (43%) |
| Stage |  |  |
| 1 | 79 (22%) | 28 (24%) |
| 2 | 35 (9.8%) | 10 (8.5%) |
| 3 | 216 (61%) | 70 (60%) |
| 4 | 27 (7.6%) | 9 (7.7%) |
| Unknown | 24 | 6 |
| Histotype |  |  |
| HGSC | 255 (67%) | 87 (71%) |
| Mucinous | 17 (4.5%) | 6 (4.9%) |
| Clear Cell | 14 (3.7%) | 6 (4.9%) |
| Carcinosarcoma | 14 (3.7%) | 3 (2.4%) |
| LGSC | 7 (1.8%) | 6 (4.9%) |
| Endometrioid | 39 (10%) | 7 (5.7%) |
| Other EOC | 30 (7.9%) | 8 (6.5%) |
| Unknown | 5 (1.3%) | 0 (0%) |
| Marital Status |  |  |
| Married | 130 (34%) | 45 (37%) |
| Single | 91 (24%) | 27 (22%) |
| Divorced | 97 (25%) | 47 (38%) |
| Widowed | 63 (17%) | 4 (3.3%) |
| Insurance |  |  |
| Yes | 268 (70%) | 84 (68%) |
| No | 112 (29%) | 38 (31%) |
| Unknown | 1 (0.3%) | 1 (0.8%) |
| Insurance Type |  |  |
| Private | 137 (36%) | 45 (37%) |
| None | 32 (8.4%) | 10 (8.1%) |
| Medicaid | 80 (21%) | 28 (23%) |
| Medicare | 98 (26%) | 20 (16%) |
| Other | 13 (3.4%) | 14 (11%) |
| Private+Medicare | 20 (5.2%) | 5 (4.1%) |
| Unknown | 1 (0.3%) | 1 (0.8%) |
| Primary Care | 338 (89%) | 103 (84%) |
| CCI |  |  |
| 0 | 138 (36%) | 43 (35%) |
| 1 | 90 (24%) | 32 (26%) |
| 2 | 61 (16%) | 19 (15%) |
| 3 | 92 (24%) | 29 (24%) |
| OC Use | 255 (67%) | 92 (75%) |
| Family History BC |  |  |
| Missing | 9 (2.4%) | 8 (6.5%) |
| No | 286 (75%) | 79 (64%) |
| Yes | 86 (23%) | 36 (29%) |
| Family History OC |  |  |
| Missing | 12 (3.1%) | 7 (5.7%) |
| No | 347 (91%) | 108 (88%) |
| Yes | 22 (5.8%) | 8 (6.5%) |
| Social Support | 39 (36, 46) | 39 (34, 46) |
| Unknown | 1 | 0 |
| Smoking Status |  |  |
| Current | 57 (15%) | 26 (21%) |
| Former | 106 (28%) | 34 (28%) |
| Never | 218 (57%) | 63 (51%) |
| Alcohol use | 151 (46%) | 60 (56%) |
| Unknown | 54 | 16 |
| Physical Activity | 87 (23%) | 39 (32%) |
| Unknown | 1 | 0 |
| Education |  |  |
| College | 66 (17%) | 32 (26%) |
| Grad School | 47 (12%) | 16 (13%) |
| HS/GED | 195 (51%) | 53 (43%) |
| Some College | 73 (19%) | 22 (18%) |
| Family Income |  |  |
| <$10,000 | 68 (18%) | 27 (22%) |
| $10,000-<$25,000 | 91 (24%) | 26 (21%) |
| $25,000-<%50,000 | 100 (26%) | 28 (23%) |
| $50,000-<$75,000 | 54 (14%) | 22 (18%) |
| $75,000-$100,000 | 35 (9.2%) | 11 (8.9%) |
| >$100,000 | 26 (6.8%) | 6 (4.9%) |
| Missing | 7 | 3 |
| Debulking Surgery | 318 (97%) | 103 (100%) |
| Unknown | 53 | 20 |
| Chemotherapy | 272 (92%) | 80 (95%) |
| Unknown | 85 | 39 |
| Imputed Debulking |  |  |
| Optimal | 265 (70%) | 90 (73%) |
| Suboptimal | 116 (30%) | 33 (27%) |
| Yost index |  |  |
| 1 | 113 (30%) | 36 (29%) |
| 2 | 63 (17%) | 18 (15%) |
| 3 | 36 (9.4%) | 18 (15%) |
| 4 | 41 (11%) | 12 (9.8%) |
| 5 | 16 (4.2%) | 5 (4.1%) |
| Unknown | 112 (29%) | 34 (28%) |
| Everyday Discrimination |  |  |
| 1 (Low) | 217 (57%) | 40 (33%) |
| 2 | 117 (31%) | 55 (45%) |
| 3 | 36 (9.4%) | 19 (15%) |
| 4 | 10 (2.6%) | 8 (6.5%) |
| 5 (High) | 0 (0%) | 1 (0.8%) |
| Unknown | 1 | 0 |
